# Venous thromboembolism incidence in postoperative breast cancer patients

**DOI:** 10.1016/j.clinsp.2023.100229

**Published:** 2023-06-10

**Authors:** Jonathan Yugo Maesaka, Yedda Nunes Reis, Livia Menezes Elias, Denise Akerman, Edmund Chada Baracat, José Roberto Filassi

**Affiliations:** a Setor de Mastologia, Divisão de Ginecologia, Instituto do Cancer do Estado de Sao Paulo, Hospital das Clinicas HCFMUSP, Faculdade de Medicina, Universidade de Sao Paulo, Sao Paulo, BR; b Disciplina de Ginecologia, Departamento de Obstetricia e Ginecologia, Faculdade de Medicina FMUSP, Universidade de Sao Paulo, Sao Paulo, BR

**Keywords:** Breast neoplasms, Venous thromboembolism, Pulmonary embolism

## Abstract

**Background:**

Venous Thromboembolism (VTE) is an important cause of morbidity in cancer patients. Breast cancer patients undergoing surgical treatment are at an increased risk of VTE. The aim of this study was to determine the frequency of VTE in patients who underwent surgery for the treatment of breast cancer and to identify the related risk factors.

**Methods:**

A historical cohort of patients at the São Paulo State Cancer Institute (ICESP) underwent surgery for breast cancer. The inclusion criteria covered patients with invasive breast cancer or ductal carcinoma *in situ* who had breast surgery anytime from January 2016 to December 2018.

**Results:**

Of the 1672 patients included in the study, 15 had a confirmed diagnosis of VTE (0.9%), and 3 of these had deep vein thrombosis (0.2%), and 12, had pulmonary thromboembolism (0.7%). Clinical and tumoral characteristics did not differ between the groups. The incidence of VTE was higher in patients who had undergone skin-sparing mastectomy or nipple-sparing mastectomy (p = 0.032). Immediate reconstruction, particularly with abdominal-based flaps (4.7%), increased VTE events (p = 0.033). Median surgical time was higher in patients with VTE episodes (p = 0.027), and total hospital length of stay increased in days (6 days vs. 2 days, p = 0.001). Neoadjuvant chemotherapy and postoperative prophylaxis with Low Molecular Weight Heparin (LMWH) were associated with lower VTE rates (0.2% vs. 1.2%, p = 0.048 and 0.7% vs. 2.7%, p = 0.039; respectively) in these patients.

**Conclusions:**

The incidence of VTE events in breast cancer patients who underwent surgery was 0.9%. Immediate reconstruction (especially with abdominal-based flaps), skin-sparing/nipple-sparing mastectomies, and longer surgeries were associated with increased risk. The LMWH postoperative prophylaxis reduced this risk.

## Highlights

• This is a pioneer study investigating VTE in breast surgery patients in Brazil.
• Patients undergoing SSM or NSM, immediate breast reconstruction – especially abdominal flap – and procedures longer than four hours had a higher incidence of VTE events.
• Neoadjuvant chemotherapy and LMWH were associated with a lower incidence of thrombotic events.

## Introduction

Venous thromboembolism (VTE) is an important cause of morbidity and mortality in cancer patients. [1] In those with breast cancer, it is the second most common cause of death. [2] Pulmonary Thromboembolism (PE), one of the forms of VTE, still represents the most frequent cause of preventable in-hospital death. [3] The economic loss associated with VTE is also substantial; the annual cost to the US healthcare system is more than 7 billion dollars. [4]

Cancer patients have a 4- to 7-fold increased risk of VTE than the general population, and the risk is higher in a few sites of primary cancer. [2,5] Furthermore, cancer patients undergoing surgical procedures are at least twice as likely to have a postoperative Deep Vein Thrombosis (DVT) and more than three times more likely to have PE when compared to noncancer patients undergoing similar procedures. [5] The identification of patients at risk for VTE and the initiation of appropriate preventive measures is, therefore, mandatory.

However, the risk of VTE and the recommendations for prophylaxis in patients undergoing surgery for breast cancer are as yet not well established. Currently, the most applied method for estimating VTE risk is the Caprini Risk Assessment Model. [3] Despite the validity of this predictive model, important clinical and therapeutic questions remain unanswered, such as the ideal duration of VTE prophylaxis.

Therefore, the authors conducted this single-institution study to determine the frequency of VTE in patients diagnosed with invasive breast cancer/Ductal Carcinoma *In Situ* (DCIS) who underwent surgery for the treatment of breast cancer. This would allow us to identify the main risk factors related to VTE in these patients.

## Methods

### Patient selection

All patients with breast cancer included in this study were treated at the São Paulo State Cancer Institute (ICESP), a leading cancer center in the country and in Latin America. They were selected from January 2016 to December 2018. Patient data were collected prospectively for inclusion in our institutional database during their treatment. The STROBE guidelines were applied to conduct this observational research. [6]

Inclusion criteria encompassed patients with invasive breast cancer or Ductal Carcinoma *In Situ* (DCIS) who had undergone breast surgery. Patients were excluded only if they missed follow-up after surgery. Breast surgery included mastectomy or lumpectomy, with or without axillary surgery, and immediate reconstruction. The incidence of VTE was the primary outcome. Two groups were formed, one with patients with VTE and another with patients without VTE. In this study, VTE was limited to Deep Venous Thrombosis (DVT) or Pulmonary Embolism (PE), confirmed by venous compression ultrasonography and contrast-enhanced tomography respectively.

The variables sourced from the collected data to undergo analysis were the following: age, sex, Body Mass Index (BMI), neoadjuvant chemotherapy, neoadjuvant hormone therapy, comorbidities, type of breast surgery, type of axillary surgery, immediate reconstruction, type of reconstruction, tumoral subtype, immunohistochemistry, the time span from diagnosis to surgery, total surgical time, VTE prophylaxis (intraoperative and postoperative), type of prophylaxis, pathological staging, the total length of stay, and need for intensive care treatment.

The authors defined systemic comorbidities as follows: i) Cardiovascular disease as a history of heart failure, coronary artery disease, previous myocardial revascularization, previous myocardial infarction or stroke; ii) Renal disease as creatinine clearance lower than 60 mL/min (calculated using the Modification of Diet in Renal Disease study equation (MDRD); [7] iii) Liver disease as impairment of hepatic function scored by the Model for End-Stage Liver Disease (MELD); [8] and iv) Vascular disease as the presence of varicose veins or any previous venous surgical manipulation.

This study was approved by the local ethics committee and is in accordance with the Declaration of Helsinki (approval number 3.402.130).

### Statistical analysis

Study data were collected and managed using the REDCap (Research Electronic Data Capture) tool. [9,10] Categorical variables are presented as counts and frequencies and were analyzed using the Chi-Square test or Fisher's exact test. Continuous variables are presented as median and Interquartile Range (IQR) and were analyzed using Mann-Whitney's *U* test. Frequency distribution tables were used to describe the results. The Chi-Square test was used to determine the difference between the two groups. Statistical significance was defined as a value of p lower than 0.05. Following univariate analysis, the authors selected all variables with a significant p*-*value for multivariate analysis using binary logistic regression. Statistical analyses were performed using SPSS 26.0 (IBM Corp., Armonk, NY, USA).

## Results

After the exclusion of 3 patients lost to follow-up, there remained 1762 patients selected from the present study's institutional database. Among these, 15 (0.9%) had a confirmed diagnosis of VTE: 3 (0.2%) had DVT and 12 (0.7%) had PE. Most patients were female, aged 40 years or older, and nonobese, and they had at least one comorbidity each.

The most common comorbidities in the VTE group were hypertension (53.3%), diabetes (33.3%), and obesity (33.3%) (p > 0.05 for all). The other comorbidities, such as hypothyroidism, liver disease, renal disease, vasculopathy, previous stroke, or coronary disease, did not differ between the groups, either (p > 0.05), (Table 1).

**Table 1:** Clinical and tumoral characteristics.

	No VTE	VTE	p
**Sex**			
Female	1630 (98.4%)	15 (100%)	1.0
Male	27 (1.6%)	0	
**Age (median, years)**	55.4	58.9	0.3
**Age**			0.95
≤40 years	224 (13.5)	1 (6.7%)	
41‒60 years	866 (52.3%)	9 (60%)	
61‒74 years	437 (26.4%)	4 (26.7%)	
> 75 years	130 (7.8%)	1 (6.7%)	
**BMI (kg/m^2^)**			0.242
Normal	465 (28.1%)	1 (6.7%)	
Overweight	594 (35.8%)	7 (46.7%)	
Obesity I	390 (23.5%)	6 (40%)	
Obesity II	141 (8.5%)	1 (6.7%)	
Obesity III	67 (4%)	0	
**Number of comorbidities**			0.572
None	459 (27.7%)	2 (13.3%)	
1	536 (32.3%)	5 (33.3%)	
2	358 (21.6%)	4 (26.7%)	
3 or more	304 (18.3%)	4 (26.7%)	
**Smoking**	238 (14.4%)	2 (13.3%)	1.0
**Hypertension**	728 (43.9%)	8 (53.3%)	0.465
**Diabetes**	282 (17%)	5 (33.3%)	0.157
**Hypothyroidism**	171 (10.3%)	4 (26.7%)	0.063
**Liver disease**	16 (1%)	0	1.0
**Renal disease**	23 (1.4%)	1 (6.7%)	0.196
**Another cancer**	67 (4%)	0	1.0
**Vasculopathy**	17 (1%)	0	1.0
**Cardiovascular disease**	51 (3.1%)	0	1.0
**Previous VTE**			1.0
Yes	31 (100%)	0	
No	1626 (99.1%)	15 (0.9%)	
**Histological subtype**			0.182
IDC	1240 (99.3%)	9 (0.7%)	
ILC	99 (100%)	0	
DCIS	158(98.1%)	3 (1.9%)	
Sarcoma/Phyllodes	18 (100%)	0	
Others	137 (97.9%)	2 (2.1%)	
**IHQ**			0.954
HR +	1005 (99.1%)	9 (0.9%)	
HR +/ HER2+	143 (99.3%)	1 (0.7%)	
HER2+	92 (98.9%)	1 (1.1%)	
Triple negative	243 (99.2%)	2 (0.8%)	
**Tumor size**			
T0	101 (100%)	0	1.00
Tis	182 (98.4%)	3 (1.6%)	0.226
< 2 cm	524 (99.6%)	2 (0.4%)	0.167
2‒5 cm	577 (98.6%)	8 (1.4%)	0.173
> 5 cm	240 (99.2%)	2 (0.8%)	1.0
**Lymph node status**			
N0‒N1	1257 (99.1%)	12 (0.9%)	1.0
N2‒N3	234 (99.6%)	1 (0.4%)	0.709
**Neoadjuvant Chemotherapy**			0.048
Yes	520 (99.8%)	1 (0.2%)	
No	1137 (98.8%)	14 (1.2%)	
**Neoadjuvant Hormonal Therapy**			1.0
Yes	61 (100%)	0	
No	1596 (99.1%)	15 (0.9%)	

VTE, Venous Thromboembolism; BMI, Body Mass Index; IDC, Invasive Ductal Carcinoma; ILC, Invasive Lobular Carcinoma; DCIS, Ductal Carcinoma *In Situ*; IHQ, Immunohistochemistry; HR, Hormone Receptor.

In the non-VTE group, Invasive Ductal Carcinoma (IDC) was the most common cancer type, while in the VTE group, other types were more frequent (p = 0.182). Neoadjuvant chemotherapy was associated with lower rates of VTE (0.2% vs. 1.2%, p = 0.048). Neoadjuvant hormonal therapy did not differ between the groups (p = 1.0). A total of 11.9% of the patients underwent surgery due to the diagnosis of DCIS, and 2.4% of them had been previously diagnosed with metastatic disease.

**Fig. 1:**
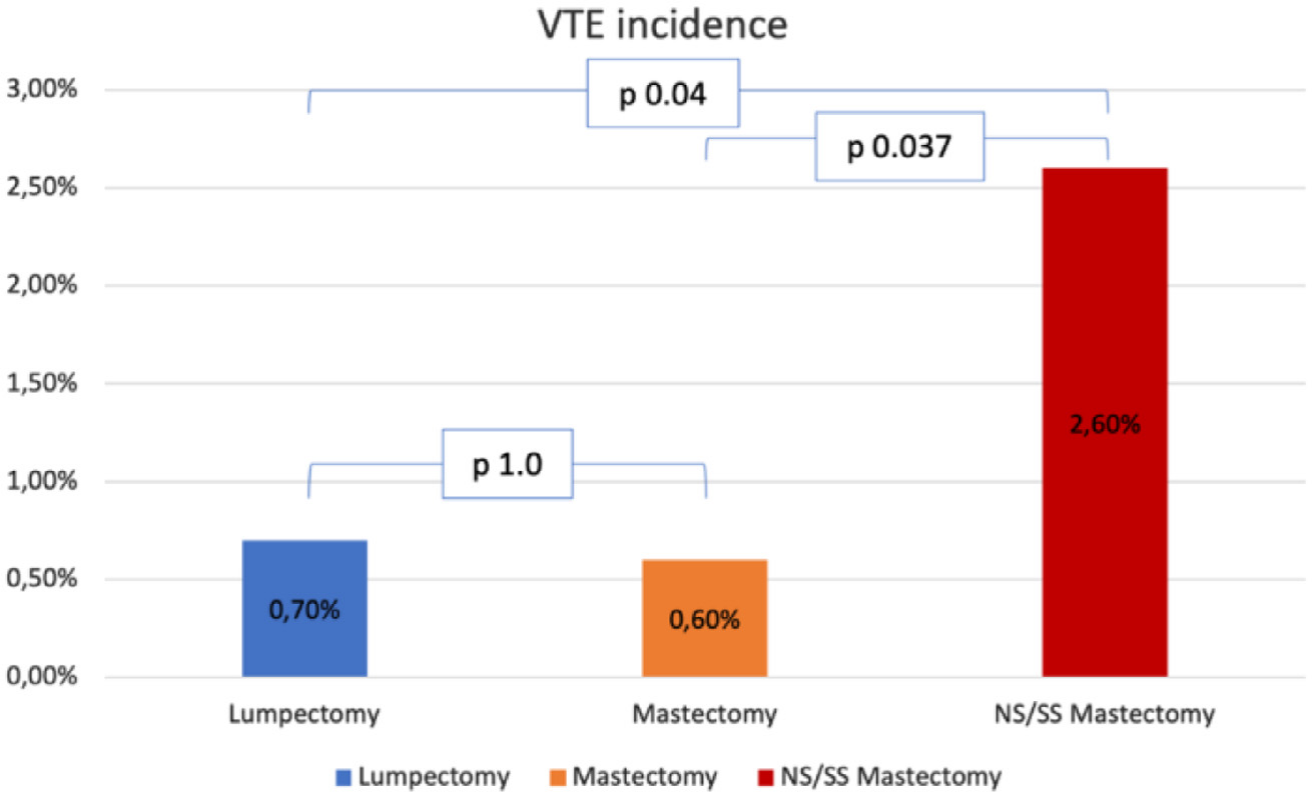
Incidence of VTE according to breast surgical procedure. VTE, Venous Thromboembolism; NS/SS, Nipple Sparing/Skin Sparing.

Surgical characteristics are described in Table 2. Roughly half of the patients (49.9%) had undergone a mastectomy, and 33.7%, had immediate reconstruction. Incidence of VTE was higher in the patients who had undergone Skin Sparing Mastectomy (SSM) or Nipple Sparing Mastectomy (NSM) than in the patients who had undergone lumpectomy or simple mastectomy (2.6% vs. 0.7% and 0.6% respectively, p = 0.032, Fig. 1). Incidence of VTE was also higher in patients who had undergone immediate reconstruction with oncoplastic surgery or implant-based reconstruction (4.7% vs. 1.6% and 0.7%, respectively, p = 0.045). Dichotomizing patients into those who had immediate reconstruction with abdominal-based flaps and those who had any other type of immediate reconstruction also showed a difference in VET events (4.7% vs. 0.9%, p = 0.014).

**Table 2:** Surgical characteristics.

	No VTE	VTE	p
**Breast surgery**			0.032
Lumpectomy	827 (99.3%)	6 (0.7%)	
Mastectomy	634 (99.4%)	4 (0.6%)	
SS/NS Mastectomy	190 (97.4%)	5 (2.6%)	
**Axillary surgery**			0.599
Sentinel Lymph node	858 (99%)	9 (1%)	
Axillary dissection	711 (99.3%)	5 (0.7%)	
No	86 (98.9%)	1 (1.1%)	
**Immediate reconstruction**			0.033
Yes	559 (98.4%)	9 9 (1.6%)	
No	1098 (99.5%)	6 (0.5%)	
**Type of reconstruction**			0.045
Implant based	299 (99.3%)	2 (0.7%)	
Oncoplastic surgery	123 (98.4%)	2 (1.6%)	
Latissimus dorsi flap	36 (100%)	0	
Abdominal flap	101 (95.3%)	5 (4.7%)	
**Hospital stay (median, days)**	2.0	6.0	0.001
**Operative time (median, minutes)**	199	293	0.027
**Total surgery time > 4 hours**			0.044
Yes	581 (98.5%)	9 (1.5%)	
No	1075 (99.4%)	6 (0.6%)	
**Intraoperative prophylaxis**			0.149
Yes	1582 (99.2%)	13 (0.8%)	
No	75 (97.4%)	2 (2.6%)	
**Compression stockings**			0.156
Yes	1580 (99.2%)	13 (0.8%)	
No	77 (97.5%)	2 (2.5%)	
**Pneumatic compression pump**			0.578
Yes	1195 (99.2%)	10 (0.8%)	
No	462 (98.9%)	5 (1.1%)	
**Postoperative prophylaxis**			0.475
Yes	1588 (99.1%)	14 (0.9%)	
No	69 (98.6%)	1 (1.4%)	
**Compression stockings**			0.921
Yes	1015 (99.1%)	9 (0.9%)	
No	642 (99.1%)	6 (0.9%)	
**Pneumatic compression pump**			0.641
Yes	12 (100%)	0	
No	1645 (99.1%)	15 (0.9%)	
**LMWH**			0.039
Yes	1511 (99.3%)	11 (0.7%)	
No	146 (97.3%)	4 (2.7%)	
**Physical therapy**			0.245
Yes	1553 (99.2%)	13 (0.8%)	
No	104 (98.1%)	2 (1.9%)	

SS, Skin Sparing; NP, Nipple Sparing; LMWH, Low Molecular Weight Heparin.

Median surgical time was higher in patients with VTE events (p = 0.027), and so was total hospital length of stay in days (6 days vs. 2 days, p = 0.001). With total surgical time over 4 hours, the incidence of VTE was significantly higher (9 cases vs. 6 cases, p = 0.044, Table 3). Intraoperative prophylaxis was not associated with lower VTE rates (p > 0.05 for all interventions), while postoperative prophylaxis with low molecular weight heparin (LMWH) was associated with a lower incidence of VTE in these patients (0.7% vs. 2.7%, p = 0.039). Median prophylaxis time was similar in both groups: 7 days (7‒9) vs. 10 days (7‒14), p. 0.138, for the non-VTE group and the VTE group respectively.

**Table 3:** Univariate analysis.

Variable	Odds Ratio (95% CI)	p-value
Neoadjuvant chemotherapy	0.15 (0.02 ‒ 1.19)	0.048
Immediate reconstruction	2.95 (1.04 – 8.31)	0.033
Type of reconstruction	5.74 (1.51 ‒ 21.74)	0.013
Total surgery time > 4 hours	2.77 (0.98 – 7.83)	0.044
Breast surgery	1.50 (0.53 ‒ 4.24)	0.437
LMWH prophylaxis	0.26 (0.08 ‒ 0.84)	0.039

LMWH, Low Molecular Weight Heparin.

In Table 4, the authors present the clinical and surgical characteristics of the patients with early VTE (< 30 days) events.

**Table 4:** Clinical characteristics of VTE patients.

Case	Age (years)	Operative time (minutes)	Neoadjuvant QT	Breast surgery	Immediate reconstruction	Smoking	Comorbidities	Clinical Staging	Prophylaxis	VTE
1	79	204	Yes	Simple mastectomy	No	No	Hypertension, obesity, Hypothyroidism, Renal disease, cardiovascular disease, hyperlipidemia	T4bN2	GCS + IPC + LMWH 14 days	DVT
				Axillary dissection						
2	72	259	No	Lumpectomy	No	No	Hypertension, hyperlipidemia	T2N0	GCS + IPC + LMWH 7 days	PE
				Sentinel lymph node						
3	58	860	No	Simple mastectomy	TRAM	No	Hypertension, obesity	T3N1M1	GCS + IPC	PE
				Axillary dissection						
				Costal arch resection						
4	49	599	No	Skin sparing mastectomy	TRAM	Yes	Hypertension, obesity	Tis	GCS + IPC + LMWH 4 days	PE
				Axillary dissection						
5	40	792	No	Skin sparing mastectomy	TRAM	No	No	Tis	GCS + IPC + LMWH 30 days	PE
				Sentinel lymph node						
6	69	203	No	Mastectomy	No	No	Hypertension, obesity, diabetes	T2N0	GCS + LMWH 4 days	PE
				Axillary dissection						
				Contralateral lumpectomy						
7	52	234	No	Simple mastectomy	Alloplastic + contralateral mammoplasty	No	Migraine	T2N1	GCS + IPC + LMWH 11 days	DVT
				Axillary dissection						
8	59	672	No	Nipple Sparing masctectomy	TRAM	No	Hypertension, diabetes	T2N0	GCS + IPC + LMWH 7 days	PE
				Sentinel lymph node						
9	47	412	No	Skin Sparing masctectomy	TRAM	No	Diabetes	T1mN0	GCS + IPC + LMWH 1 days	TPE
				Sentinel lymph node						
10	58	430	No	Lumpectomy Sentinel lymph node	Local flap + lymph node transplantation	No	No	T1N0	GCS + IPC + LMWH 7 days	DVT

GCS, Graduated Compression Stockings; IPC, Intermittent Pneumatic Compression.

Fig. 1 shows the VTE incidence according to breast surgical procedures. comparing Each type is compared individually. No difference in VTE incidence was found between lumpectomy and mastectomy (p = 1.0). On the other hand, Incidence in the NS/SS mastectomy group was higher than in the other two groups: comparison with the lumpectomy and the mastectomy groups yielded p = 0.04 and p = 0.037 respectively.

## Discussion

It is well known that cancer patients have a higher frequency of VTE events and that surgical procedures increase the risk. The present results show that the patients who underwent SSM or NSM or immediate breast reconstruction, especially abdominal flap, had a higher incidence of VTE events and that procedures longer than four hours also increased VTE events. Contrariwise, neoadjuvant chemotherapy, and LMWH were associated with lower incidence of thrombotic events. This finding might be related to the lower surgical complexity and shorter duration of the surgery, as neoadjuvant chemotherapy patients could have smaller lesions and thus could undergo minor surgeries more frequently instead. Other factors impacting the results might be related to improved control of personal comorbidities and to habits such as physical activity and no smoking.

Despite the well-established use of predictive models, [3] assessment of VTE risk and initiation of preventive measures are not firmly implemented in the routine of all institutions. In a UK survey of 126 surgeons performing breast surgery, 30% of them did not routinely perform thromboembolism prophylaxis, for they considered breast cancer patients to be at low risk for thromboembolic complications. The estimated incidence of postoperative VTE in the study was less than 1%. [11] In a recent prospective Japanese cohort, the prevalence of VTE at treatment initiation according to cancer type was reported. Breast cancer had the lowest prevalence among solid tumors (2%), while pancreatic cancer had the highest (8.5%). On subanalysis, VTE prevalence appeared to increase as the cancer stage increased, reaching 4% in stage IV breast cancer patients. [12]

Another issue is the optimal duration of prophylaxis. In the present study, 10 out of the 15 VTE events (66%) occurred in the first 30 days after surgery. In a cohort study using English healthcare data on 13,202 patients after surgery, the risk significantly increased in the first month (HR = 2.2; 95% CI 1.4‒3.4; AR = 23.5; reference group, no surgery), but not after the first month. [13] In another retrospective cohort of 52,457 women surgically treated for breast cancer, 395 (0.8%) were diagnosed with VTE when surgery was performed during their index hospitalization or within 90 days of discharge. Most of the VTE cases (67.1%) were diagnosed after discharge from the hospital. The mean and median number of days between hospital discharge and VTE diagnosis was 26.2 and 13.0 days respectively. [3]

Risk factors for VTE other than cancer's hypercoagulability state include surgery, chemotherapy, hormone therapy, radiation therapy, and the use of central catheters. [14] In breast cancer patients, still, other factors may influence the risk of VTE. For example, the chemotherapy/hormone therapy combination increases the incidence of VTE by 9%, while hormone therapy alone revealed a 2- to 5-fold increased risk. [14] Other studies found that chemotherapy increased the risk of VTE between 2 and 10 times. [13,15,16] In contrast, the present findings showed that patients who had received chemotherapy had fewer VTE events.

Relatively longer surgeries (> 4 hours) were associated with an increased risk of VTE (OR = 2.77, 95% CI 0.98–7.83, p = 0.044) in the present study. Tran et al. in a case series also found that surgeries lasting longer than 3 hours had an increased risk of VTE (OR = 4.36, 95% CI 3.02‒6.30, p < 0.001). [2] Immediate reconstruction usually increases surgical time, and thus it was also associated with more cases of VTE events in the present study. Castaldi et al. in a retrospective study using a French database analyzed 40 986 lumpectomies and 35 909 mastectomies, and VTE was found in 172/76 895 patients (0.2%). In these patients, mastectomy with immediate autologous breast reconstruction comprised the major risk (OR = 8.792, p < 0.001; 95% CI 3.618‒21.367). [17]

Londero et al. analyzed a cohort of 5039 patients who underwent breast surgery and were followed up for a median of 75 months. Surgery-related VTE events and their distribution were considered for the first three months after the procedure. Interestingly, the cumulative incidence of VTE among women with benign histology or DCIS was 0%, and among those with invasive breast cancer, it was 0.4% (95% CI 0.19‒0.61%) (p < 0.05). [18] In the present sample, 3 patients with DCIS (1.6% of all DCIS cases) presented VTE events: one patient 76 days after surgery; and the other two, 3 and 4 days after the procedure respectively. The latter two patients underwent immediate reconstruction with abdominal-based flaps. Nevertheless, no statistical difference in VTE incidence was found according to the histological subtype in the present analyses.

Abdominal flaps are important options in breast reconstruction procedures, offering definitive closure of the surgical wound with good aesthetic results for the patient. However, their execution requires longer surgery time and large areas of tissue mobilization. Momeni et al. reported that the prophylactic use of low-molecular-weight heparin was effective in preventing pulmonary thromboembolism without increasing problematic bleeding-related complications for patients of immediate TRAM flap breast reconstruction surgery. [3] Only a few reports have assessed the incidence of pulmonary thromboembolism in TRAM flap surgery. The incidence is reported to vary from 0.7 to 18.8 percent. 19, 20, 21, 22 In the present study, abdominal flap reconstruction was associated with an increased risk of VTE.

Apart from the importance of VTE as a mortality-associated factor in breast cancer patients, the costs related to prophylactic measures for VTE for the health system drive the need for adequate tools to help screen the patients who effectively need prophylaxis. Such costs may also impel the escalation of prophylactic measures for VTE. Andtbacka et al. reported a rate of 0.16% per breast cancer surgery with no related deaths, stating that systemic VTE prophylaxis is not indicated for this group of patients. [5] The present study is a retrospective cohort, presenting real-world evidence of VTE aspects in breast cancer surgery treatment. The authors believe that Andtbacka's research findings and ours are compatible and can be applied in other centers.

Efforts were made to minimize the risk of bias: all patients operated on during the study period were evaluated; as part of the institutional protocol, cases that raised any clinical suspicions were diagnosed as VTE only when they met the diagnostic requirements; and most of the clinical variables frequently associated with the disease in other publications were included in the present analyses.

The present study has several limitations. As this is a retrospective analysis, some caution is needed when interpreting the results. Also, the low frequency of events (only 15 VTE) limited statistical multivariate analysis. Finally, some events occurred 30 days after the surgery, and thus they might not have been related to the surgical procedure, but rather to the cancer itself. However, the strengths of the present study far surpasses its limitations. First, it is a pioneer study investigating VTE in breast surgery patients in Brazil, a population with higher rates of ethnic and genetic miscegenation than the European, North American, or Asian populations in general. Second, the patients were treated at a national reference center for oncology, receiving the best clinical and surgical care available in Brazil. Patient care was homogeneous throughout the present sample, with guideline-oriented treatments being adopted whenever possible. All patients were closely monitored, and all events were thoroughly investigated.

## Conclusion

The incidence of VTE events in breast cancer patients who underwent surgery was 0.9% in the historical cohort. Immediate reconstruction (especially with abdominal-based flaps), skin-sparing/nipple-sparing mastectomies, and longer surgeries were associated with an increased VTE risk. The LMWH as postoperative prophylaxis reduced the risk.

## Authors’ contributions

J.Y.M., E.C.B., J.R.F. – conception, design. J.Y.M., L.M.E., D.A. – acquisition of data. J.Y.M., Y.N.R. – data analysis. J.Y.M., Y.N.R. – draft the article. All authors approved the final version.

## Declaration of Competing Interest

The authors declare no conflicts of interest.
